# Extensive Peritoneal and Pelvic Granulomatous Inflammation Due to Syphilis Infection Manifesting as Primary Infertility: A Case Report

**DOI:** 10.7759/cureus.70764

**Published:** 2024-10-03

**Authors:** Elias Tsakos, Emmanouil M Xydias, Apostolos C Ziogas, Katerina Zarampouka, Spyridon Gerou, Nikolaos Tsagias, Kanelina Bimpa

**Affiliations:** 1 Department of Obstetrics and Gynecology, EmbryoClinic IVF, Thessaloniki, GRC; 2 Department of Obstetrics and Gynecology, University of Thessaly, Larissa, GRC; 3 Department of Pathology, Istodierevnitiki SA, Thessaloniki, GRC; 4 Department of Biopathology, Analysi Iatriki SA, Thessaloniki, GRC; 5 Department of Breast Surgery, EmbryoClinic IVF, Thessaloniki, GRC

**Keywords:** assisted reproduction (art), chronic granulomatous inflammation, infertility, intraabdominal adhesions, laparoscopy, pelvic inflammatory diasease, syphilis, tubal inflammation, tubal obstruction

## Abstract

Infertility affects millions of couples worldwide and can result from various factors, including sexually transmitted infections. Although syphilis is known to contribute to a small number of infertility cases through chronic pelvic inflammatory disease, which ultimately impairs fertility, detailed descriptions of such cases are limited. In this report, we present a case of primary infertility caused by extensive peritoneal granulomatous inflammation, adhesions, and tubal obstruction resulting from syphilis.

## Introduction

Infertility, particularly primary infertility, has become a significant issue in the modern world, affecting an estimated one in six couples who experience difficulties conceiving spontaneously [1]. Various factors contribute to this condition, including impaired sperm function, tubal obstruction, ovulatory disorders, and complications arising from malignancy or chemotherapy [2,3]. Tubal factor infertility accounts for approximately 30% of these cases, primarily due to sexually transmitted infections, especially those caused by *Chlamydia trachomatis *and *Neisseria gonorrhoeae *[4]. These bacteria ascend from the primary site of infection in the external genitalia through the epithelium of the genitourinary tract, ultimately reaching the internal genitalia and leading to conditions such as salpingitis or, in more severe instances, pelvic inflammatory disease (PID) [5]. Recurrent acute inflammation or chronic, untreated salpingitis - often asymptomatic and underdiagnosed - can result in chronic pelvic inflammation and the formation of tubal and pelvic adhesions, leading to tubal obstruction and subsequent infertility [4,5].

While infections caused by* C. trachomatis *and *N. gonorrhoeae *account for the majority of cases, several other pathogens have been implicated in infertility, as indicated by serological studies. Notably, *Mycoplasma genitalium *and *Trichomonas vaginalis *have emerged as significant contributors [4]. In regions with higher prevalence, *Mycobacterium tuberculosis *has also been linked to impairments in tubal anatomy and function, resulting in an increased incidence of ectopic pregnancies [6]. Additionally, some studies have suggested associations with common urogenital pathogens such as *Mycoplasma hominis *and *Ureaplasma urealyticum*, although the evidence is less conclusive [4]. Among the less common pathogens associated with PID is *Treponema pallidum*, the causative agent of syphilis, which has been identified in serological screenings of patients with a history of PID [7]. It is rare for syphilis to directly impair fertility without other notable clinical manifestations; its more significant risks regarding childbearing typically arise after conception, including recurrent miscarriages and increased neonatal morbidity and mortality [8].

In this report, we present the case of a woman seeking assisted reproductive technology (ART) services due to primary infertility, ultimately attributed to severe chronic granulomatous peritoneal inflammation resulting from tertiary syphilis.

## Case presentation

A 30-year-old woman visited our clinic with her husband, seeking ART services due to primary infertility lasting seven years. The couple had previously sought ART services in the UK four years prior. Fertility assessment examinations showed decreased anti-Müllerian hormone levels of 1.16 ng/ml, and fallopian tube patency assessment indicated bilateral obstruction. Two years later, they underwent three in vitro fertilization (IVF) cycles, resulting in five viable embryos - three transferred in a fresh cycle and two in a frozen cycle (cryopreserved for later transfer following a preparation protocol). Four of these attempts resulted in implantation failure, while one of the frozen embryo transfer attempts (the fourth of the total five) resulted in a biochemical pregnancy and subsequent loss at the fifth week of gestation.

Regarding her medical history, the patient reported a previous syphilis *(T. pallidum*) infection, which she stated was treated with several courses of penicillin. Her gynecological history included regular 28-day cycles, with occasional two-day deviations. Her husband, 37 years old, had no notable medical history and had undergone a normal semen analysis a year before their presentation to our clinic.

**Figure 1:**
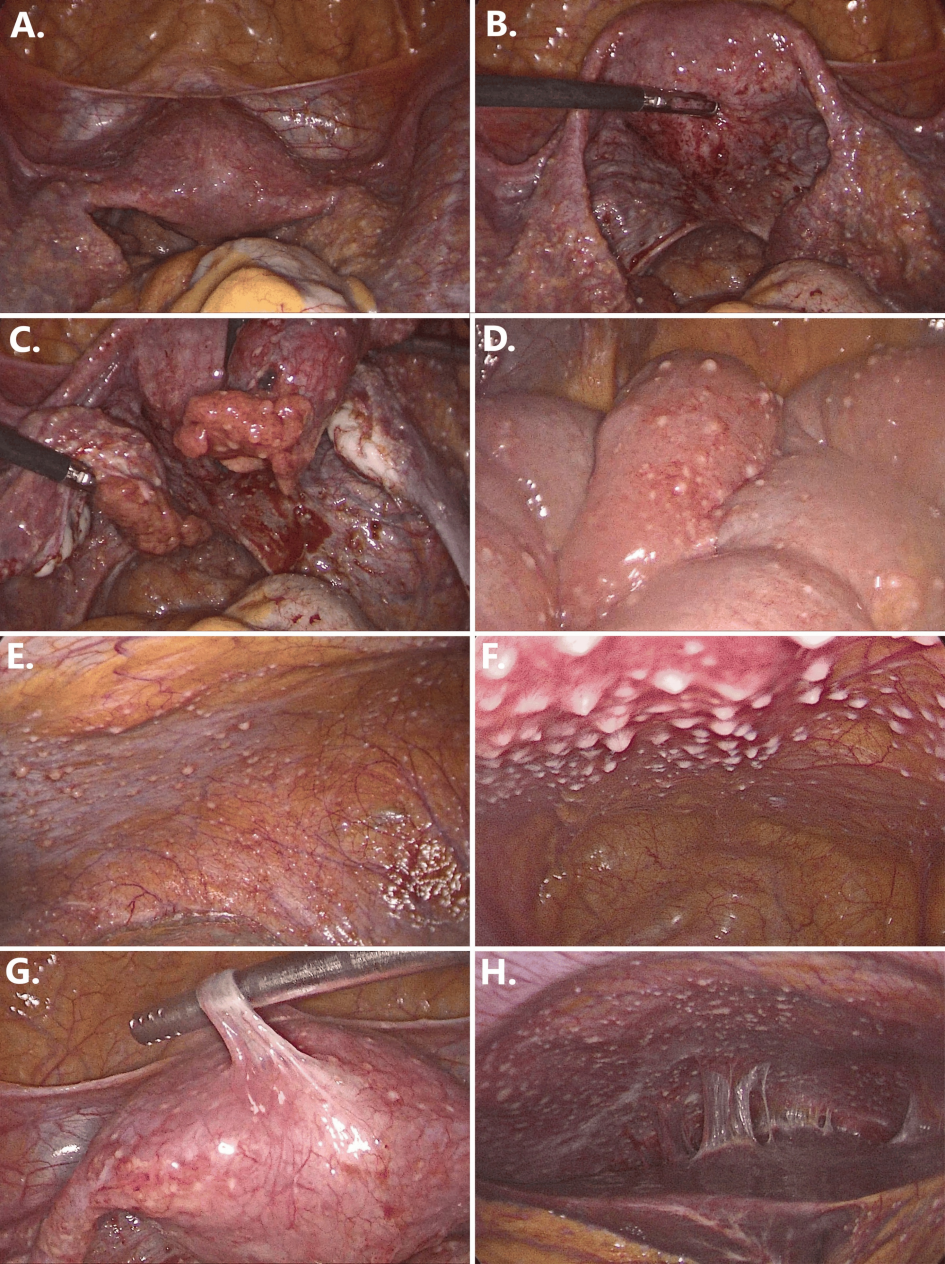
Endoscopic images of the peritoneal cavity obtained during the initial laparoscopic assessment (A) Multiple granulomas on the anterior aspect of the uterus and broad ligament. (B) Multiple granulomas on the posterior aspect of the uterus. (C) Multiple, easily ruptured granulomas on the adnexa. (D) Multiple granulomas on the intestinal surface, accompanied by intestinal distention. (E, F) Multiple granulomas surrounded by tissue inflammation on the parietal peritoneal surface. (G) Adhesions on the surface of the uterus. (H) Peri-hepatic adhesions.

In light of the multiple implantation failures and the previous history of fallopian tube obstruction, the couple was offered a combined hysteroscopic and laparoscopic assessment of the peritoneal cavity. Hysteroscopy revealed slightly hyperemic endometrial epithelium and confirmed bilateral tubal ostia occlusion. During the initial laparoscopic inspection, most intraperitoneal tissues exhibited characteristics of chronic inflammation, including pseudo-membranes and adhesions throughout the entire cavity (peri-uterine, peri-hepatic, etc.), as well as extensive granuloma-like lesions that diffusely spread. The uterus was of normal size but showed signs of chronic inflammation and granulomas. The fallopian tubes exhibited severe bilateral inflammation, were largely distended, and displayed significant distortion of their local anatomy. These observations were consistent with chronic PID with extra-pelvic spread to the entire peritoneum. The most relevant findings from the laparoscopic inspection are presented in Figure 1.

**Figure 2:**
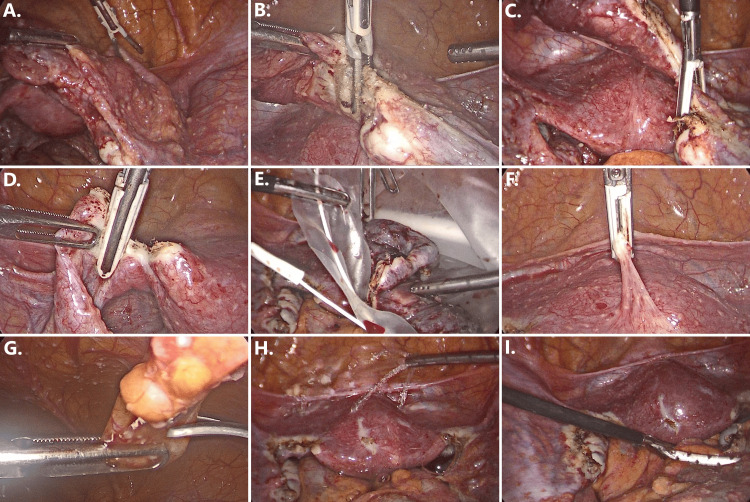
Step-by-step endoscopic images illustrating the laparoscopic salpingectomy procedure (A) Identification of the salpinx. (B) Desiccation and (C) incision at the distal (ovarian) end. (D) Desiccation and incision at the proximal (uterine) end. (E) Excision and removal of both tubes via surgical bag. (F) Adhesiolysis of uterine and peri-uterine adhesions. (G) Biopsy collection from macroscopically visible granulomas of the peritoneum. (H) Hemostasis and irrigation. (I) Abdominal drain placement.

Following the inspection, bilateral salpingectomy was performed, and the inflamed fallopian tubes were removed using surgical bags (Figure 2). Additionally, biopsies were collected from the granulomas present on the surface of the uterus and peritoneum and sent for histopathological analysis. The operation was completed within 80 minutes, with negligible blood loss, and the patient made a swift recovery without any complications.

Histopathologic examination of endometrial samples collected during hysteroscopy revealed significant disturbances in tissue architecture, characterized by dense infiltration of lymphocytes and plasmocytes, along with the formation of granulomas. These granulomas were predominantly small, consisting of epithelioid mastocytes and varying numbers of multinucleated giant cells of both the Langhans and foreign-body types. No cellular necrosis was noted.

Examination of the peritoneal biopsy material showed numerous small granulomas, similarly composed of epithelioid mastocytes and multinucleated giant cells, without evidence of cellular necrosis. Lymphocyte and plasmocyte infiltration surrounded the granulomas.

**Figure 3:**
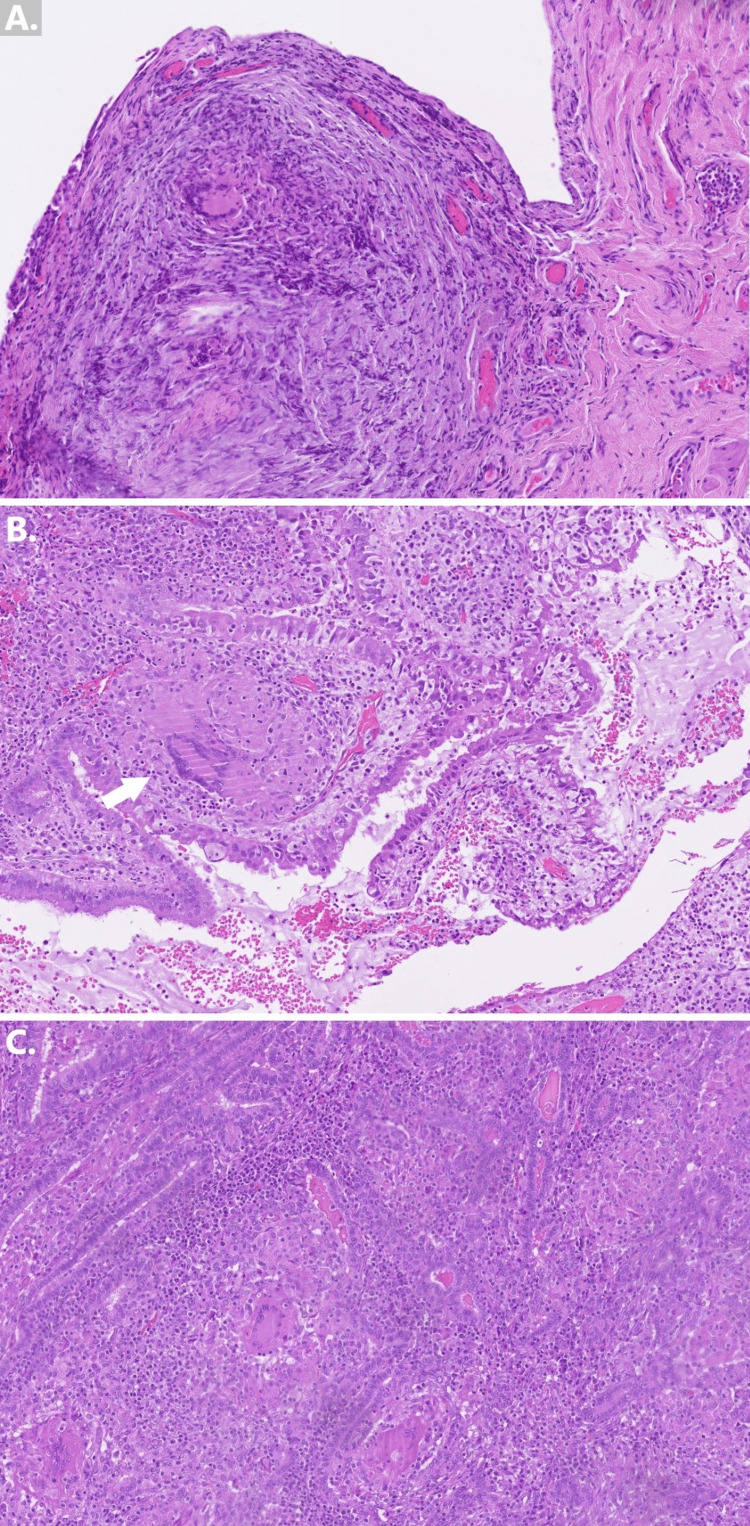
Histopathologic assessment of the biopsied material stained with H&E, observed at 10X magnification (A) Noncaseating granulomas in the endometrium (specimen of curettage). (B) Noncaseating granuloma in a sample of the peritoneum with crashing lesions. Langhans giant cell is observed (arrow). (C) Noncaseating granulomas in the mucosa of the fallopian tube.

Finally, histopathologic examination of the removed fallopian tubes revealed extensive damage to the salpingeal mucosa. In the more well-preserved segments, there was significant disruption of normal tissue architecture, with marked stroma broadening and dense infiltration by lymphocytes and plasmocytes. Granulomas, resembling those previously described, were also identified, but no necrosis or granulomas within vascular walls were observed. Ziehl-Neelsen staining was negative. The observed lesions indicated granulomatous inflammation of the endometrium, fallopian tubes, and peritoneum. The most notable histopathological findings are illustrated in Figure 3.

Following histopathological assessment, a laboratory investigation was conducted to further evaluate the patient and ascertain the origin of the granulomas, as well as the activity of her known syphilis infection. Examination of sputum and peripheral blood tested negative for *M. tuberculosis*. However, peripheral blood tests were positive for *T. pallidum *infection, indicating incomplete initial treatment and suggesting chronic, asymptomatic PID attributed to syphilis.

The patient was referred to a specialized venereal disease center, where serological tests for syphilis were repeated three and five months postoperatively. All relevant laboratory investigations are summarized in Table 1. A serofast status for syphilis was confirmed; therefore, no additional antibiotic treatment was prescribed. At the time of writing, the patient and her partner had been cleared to proceed with ART, although they chose to postpone the process for nonmedical reasons.

**Table 1:** Summary of all laboratory and molecular investigations conducted following laparoscopy ELISA: enzyme-linked immunosorbent assay; FTA-ABS: fluorescent treponemal antibody absorption; FU: follow-up; IGRA: interferon-gamma release assay; PCR: polymerase chain reaction; RPR: rapid plasma reagin; RT-PCR: real-time polymerase chain reaction; TIA: turbidimetric immunoassay; TPHA: *Treponema pallidum* hemagglutination assay; VDRL: venereal disease research laboratory test

Time	Sample	Examination	Results	Normal values
Within one month of laparoscopy	Sputum	*Mycobacterium tuberculosis *complex PCR	Negative	Negative
	Sputum	*Mycobacterium avium *complex PCR	Negative	Negative
	Sputum	*Treponema pallidum *PCR	Negative	Negative
	Endometrium	*T. pallidum *RT-PCR	Negative	Negative
	Peripheral blood	IGRA (QuantiFERON®-TB Gold Plus)	0.28	<0.35 IU/mL, Negative
	Peripheral blood	VDRL	Positive	Negative
	Peripheral blood	RPR	Positive	Negative
	Peripheral blood	FTA-ABS (*T. pallidum*)	>45.0	<1.0 IV, Negative
	Peripheral blood	TPHA	Positive	Negative
Three- and five-month FU	Peripheral blood	Syphilis TP Latex assay	Positive	Negative
	Peripheral blood	TPHA	Positive	Negative
	Peripheral blood	Syphilis total antibodies EIA	Positive	Negative
	Peripheral blood	Syphilis IgM ELISA	Negative	Negative
	Peripheral blood	RPR	1:8 Positive	Negative

## Discussion

The present case illustrates an uncommon manifestation of syphilis in the context of infertility treatment and ART. Syphilis ranks as the third most prevalent bacterial sexually transmitted infection, with an average incidence of seven reported cases per 100,000 individuals in Europe in 2021 [9,10]. Clinical manifestations of the disease typically follow a cyclical pattern, characterized by symptomatic and asymptomatic periods. During the early phase, approximately one year after the initial infection, manifestations can be classified into primary and secondary stages, with transient stages occurring in between [9]. The primary stage typically presents as a solitary, ulcerated, painless lesion at the site of inoculation, appearing about three weeks post-transmission [11]. The secondary stage arises four to 10 weeks after initial transmission and is characterized by generalized lymphadenopathy, accompanied by malaise and/or fever [11]. Cutaneous manifestations are common at this stage, including macular or papulosquamous exanthema located on the trunk and extremities, often involving the palms and soles [9,11]. Additionally, condyloma latum in both genital and extra-genital areas, alopecia, and mucosal lesions may occur during the secondary stage, although more atypical presentations, such as nodular or pustular ulcerative syphilis, are less frequent [11,12]. Symptoms at the tertiary stage of syphilis vary depending on the affected system (skin, neurological, or cardiovascular) due to the granulomatous reaction [11].

In terms of fertility, syphilis is recognized as a rare but plausible cause of PID [7], a condition linked to pelvic inflammation, adhesion formation, tubal obstruction, and ultimately infertility. PID can extend beyond the upper reproductive tract into the peritoneal cavity, leading to complications such as peritoneal adhesions, abscesses, and perihepatic adhesions, the latter of which may manifest as Fitz-Hugh-Curtis syndrome in certain cases [13]. PID is often characterized by pelvic pain, abnormal vaginal discharge, and other symptoms, but it can also be asymptomatic for extended periods, with infertility being the sole indication of an underlying abnormality [14]. Our observations in this case align with the existing literature, noting peritoneal inflammation and adhesions that were asymptomatic except for the couple’s infertility. However, this report is, to our knowledge, the first to document an intra-peritoneal granulomatous reaction through surgical images and histopathology, identifying syphilis as the sole implicated pathogen.

Beyond PID, manifestations of syphilis in the abdominal and pelvic regions have been previously reported, including intra-abdominal pain due to vascular involvement [15], syphilitic gastritis presenting with alimentary tract symptoms [16], and hepatic masses that mimic metastatic cancer [17]. In these three cases, however, syphilis infection was localized, contrasting with the diffuse granulomatous inflammation of the peritoneum observed in our case. Previous reports have indicated uterine and salpingectomy involvement, such as in the study by Lawlor and Rodan in 1953 [18], which similarly noted salpingeal adhesions and dense endometrial stroma infiltrated by plasma cells and lymphocytes. This earlier case also described a patient who remained childless despite attempts at conception, suggesting a commonality with our case regarding infertility [18]. Nevertheless, the presence of two gummatous uterine masses and histologically confirmed perivascular infiltration would be more indicative of tertiary syphilis, whereas our case presented without vascular or neurological symptoms and no noted perivascular infiltration, suggesting intraperitoneal rather than vascular dissemination of the disease.

Regarding fertility outcomes and ART in such cases, Lin et al. [19] concluded that syphilis seropositivity does not significantly affect fertilization, pregnancy, miscarriage, or live birth rates if proper treatment protocols are followed. Furthermore, no significant differences were noted in neonatal outcomes, such as birth weight, preterm birth, or anomalies diagnosed at birth [19]. A similar study by Miao et al. [20] explored the impact of multiple embryo transfers on IVF outcomes, creating subgroups based on the serostatus of partners. In the subgroup with a healthy male partner and a seropositive female partner, relevant to our case, embryo implantation and clinical pregnancy rates were similar; however, live birth rates were significantly lower, and miscarriage rates were higher compared to healthy controls (50.0% vs. 71.3% and 26.7% vs. 7.8%, respectively) [20]. This contrasts with Lin et al.’s findings. Notably, in the subgroup undergoing three or more frozen embryo transfers, no statistically significant differences emerged between the two groups, aligning with Lin et al.’s results. Although these data are limited due to the overwhelming number of seronegative patients in each study, the findings are encouraging, suggesting that repeated attempts may enhance the likelihood of successful implantation and pregnancy.

## Conclusions

Syphilis continues to be a significant concern, especially in the context of infertility. Chronic asymptomatic PID resulting from syphilis can go undiagnosed for many years, leading to severe complications, such as widespread granulomatous inflammation of the peritoneum and impaired fallopian tube function, as illustrated in this case. Nevertheless, with the advancements in ART, achieving conception and delivering a healthy baby is more than possible for these women. Reproductive specialists must remain vigilant in detecting, treating, and supporting patients with syphilis on their journey to parenthood.
